# A Meta-Analysis of Interleukin-8 -251 Promoter Polymorphism Associated with Gastric Cancer Risk

**DOI:** 10.1371/journal.pone.0028083

**Published:** 2012-01-18

**Authors:** Huiping Xue, Jianjun Liu, Bing Lin, Zheng Wang, Jianhua Sun, Gang Huang

**Affiliations:** 1 Division of Gastroenterology and Hepatology, Renji Hospital, Shanghai Jiaotong University School of Medicine, Shanghai Institution of Digestive Disease, Key Laboratory of Gastroenterology & Hepatology, Ministry of Health, Shanghai Jiaotong University, Shanghai, People's Republic of China; 2 Department of Nuclear Medicine, Renji Hospital, Shanghai Jiaotong University School of Medicine, Shanghai Jiaotong University, Shanghai, People's Republic of China; 3 Division of Nutrition, Zhongshan Hospital, Fudan University School of Medicine, Fundan University, Shanghai, People's Republic of China; 4 Department of General Surgery, Renji Hospital, Shanghai, People's Republic of China; National Cancer Center, Japan

## Abstract

**Background:**

Potential functional allele A/T single nucleotide polymorphism (SNP) of Interleukin 8 (IL-8) promoter -251has been implicated in gastric cancer risk.

**Methods:**

We aimed to explore the role of A/T SNP of IL-8 -251 in the susceptibility to gastric cancer through a systematic review and meta-analysis. Each initially included article was scored for quality appraisal. Desirable data were extracted and registered into databases. Eighteen studies were ultimately eligible for the meta-analysis of IL-8 - 251 A/T SNP. We adopted the most probably appropriate genetic model (codominant model). Potential sources of heterogeneity were sought out via stratification and sensitivity analyses, and publication biases were estimated.

**Results:**

Between IL-8 -251 AA genotype with gastric cancer risk, statistically significant association could be noted with overall gastric cancer, evidently noted in Asians, witnessed in high quality subgroup, and apparently noted in intestinal-type gastric cancer.

**Conclusions:**

Our meta-analysis indicates that IL-8 -251 AA genotype is associated with the overall risk of developing gastric cancer and may seem to be more susceptible to overall gastric cancer in Asian populations. IL-8 -251 AA genotype is more associated with the intestinal-type gastric cancer. IL-8 -251 AA genotype is not associated with *Helicobacter Pylori i*nfection status in our meta-analysis.

**Impact:**

The analyses suggest that IL-8 -251 AA genotype may be an important biomarker of gastric cancer susceptibility for Asians, especially for Chinese Han population, the assumption that needs to be further confirmed in future well-designed studies in China.

## Introduction

Nowadays, worldwide gastric cancer incidence has decreased but its mortality still ranks second [1]. In the midwestern rural areas of China [2], gastric cancer still constitutes one of the most lethal malignancies. As is widely known, infectious, dietary, environmental, and genetic factors are implicated in gastric carcinogenesis, but only a minority of persons exposed to risk factors such as *Helicobacter pylori* infection ultimately develop gastric cancer [3], which implies that host genetic susceptibility plays an important role in developing gastric cancer. Such various susceptibilities could be explained, in part, by single nucleotide polymorphisms (SNPs) of susceptible genes [4], [5].

IL-8, one of key members of the human α-chemokine subfamily, acts as a potent chemoattractant and activator of neutrophils [6], [7]. Highly expressed levels of IL-8 mRNA and protein were found in gastric cancer cells [8], [9]. It has recently been suggested that IL-8 is closely related to the tumorigenesis, angiogenesis, adhesion, invasion or metastasis of cancer [10]–[14]. The IL-8 gene, located on chromosome 4q12-21, is 5.2 kb long and contains four exons and three introns. In the year 2000, Hull et al. reported a single A/T SNP at position -251 numbering from the transcription start site in the proximal promoter region and found that the IL-8 -251A allele tended to be associated with increased IL-8 production [15]. Thus, it could be extrapolated that IL-8 -251A allele may increase the risk of developing cancer through the elevation of its IL-8 expression.

In 2004, Savage et al. published the first study indicating that IL-8 -251A allele is associated with an increased risk for gastric cardia cancer [16]. Since then, researchers have consecutively reported associations of IL-8 -251 A/T SNP with the susceptibility to gastric cancer, but with mixed or conflicting results [17]–[34]. Up to now, there have been five relevant published meta-analysis articles focusing on IL-8 -251 A/T SNP [35]–[39], among which one [35] was published in Chinese. Two articles were dealt with the meta-analyses on overall cancer susceptibilities rather than gastric cancer susceptibility per se in details [38], [39]. Unfortunately, those five meta-analyses all failed to adopt the most likely appropriate genetic model, and thus the authentic values of statistical results could be compromised.

Accordingly, the aim of our meta-analysis was to explore, using the most appropriate genetic model, the role of Il-8 -251 A/T SNP in the risk of developing gastric cancer and to identify possible sources of heterogeneity among the eligible studies.

## Materials and Methods

### Search Strategy

A systematic literature search was performed for articles regarding IL-8 -251 A/T SNP associated with the gastric cancer risk. The MEDLINE, EMBASE databases, Chinese National Knowledge Infrastructure (CNKI), Web of Science, and BIOSIS databases were used simultaneously with the combination of terms “Interleukin 8”, “IL-8”, “interleukin”, or “cytokine”; “gene”; “polymorphism”, “variant”, or “SNP”; and “gastric cancer”, “gastric carcinoma”, “diffuse gastric cancer” or “stomach cancer” from January 2000 to January 2011. The search was performed without any restriction on language. The scope of computerized literature search was expanded according to the reference lists of retrieved articles. The relevant original articles were also sought manually.

### Study Selection

Studies concerning the association of IL-8 -251 A/T SNP with the risk of developing gastric cancer were included if the following conditions were met: (i) any study described the association of IL-8 -251 A/T SNP with gastric cancer; (ii) any study reported the numbers of both controls and gastric cancer cases; (iii) results were expressed as odds ratio (OR) with 95% confidence intervals (CI); and (iv) studies were case-control or nested case-control ones.

### Methodological Quality Appraisal

To identify high-quality studies, we mainly adopted predefined criteria for Quality Appraisal initially proposed by Thakkinstian et al. [40], adapted by Camargo et al. [41], and refined by Xue et al. [5], [42]. The criteria (seen in **Table S1** online) cover credibility of controls, representativeness of cases, consolidation of gastric cancer, genotyping examination, and association assessment. Methodological quality was independently assessed by two investigators (J. Liu and B. Lin). Disagreements were resolved through discussion. Scores ranged from the lowest zero to the highest ten. Articles with the score lower than 6.5 were considered “low-or-moderate quality” ones, whereas those no lower than 6.5 were thought of as “high quality” ones.

### Data Extraction

The following data from each article were extracted: authors, year of publication, country, ethnicity of participants (categorized as Caucasians, Asians, etc.), study design, source of controls, number of controls and of cases, genotyping method, distribution of age and gender, Lauren's classification (intestinal, diffuse, or mixed), anatomical classification (cardia or non-cardia cancer) and *Helicobacter Pylori* infection status.

The data were extracted and registered into two databases independently by two investigators (J. Liu and B.Lin) who were blind to journal names, institutions or fund grants. Any discrepancy between these two investigators was resolved by the investigator (H. Xue), who participated in the discussion with them and made an ultimate decision.

### Statistical Analysis

All statistical analyses were performed using STATA statistical software (Version 10.1, STATA Corp, College Station, TX). Two-sided Ps<0.05 were considered statistically significant.

Hardy-Weinberg equilibrium (HWE) in controls was calculated again in our meta-analysis. The chi-square goodness of fit was used to test deviation from HWE (significant at the 0.05 level).

Odds ratios (OR) and 95% confidence intervals (95% CI) were used to assess the strength of associations between IL-8 -251 A/T SNP and gastric cancer risk. OR_1_, OR_2_, and OR_3_ regarding IL-8 -251 A/T SNP were calculated for genotypes AA versus TT, TA versus TT, and AA versus TA, respectively.

The above pairwise differences were used to determine the most appropriate genetic model. If OR_1_ = OR_3_≠1 and OR_2_ = 1, a recessive model is suggested. If OR_1_ = OR_2_≠1 and OR_3_ = 1, a dominant model is implied. If OR_2_ = 1/OR_3_≠1 and OR_1_ = 1, a complete overdominant model is suggested. If OR_1_>OR_2_>1 and OR_1_>OR_3_>1, or OR_1_<OR_2_<1 and OR_1_<OR_3_<1, a codominant model is indicated [43]. If a dominant model was indicated, the original grouping was collapsed and the new group of A carriers (AA plus TA) was compared with TT genotype; if a recessive model was suggested, AA was compared to the group of TT plus TA; if a complete overdominant model was implied, the group of AA plus TT was compared with TA; or if a codominant model was insinuated, AA was compared with TA and with TT, respectively.

The Q statistic was used to test for heterogeneity among the studies included in the meta-analysis. A fixed-effects model, using Mantel–Haenszel (M-H) method, was employed to calculate the pooled ORs when homogeneity existed on the basis of Q-test p value no less than 0.1.By contrast, a random-effects model, using DerSimonian and Laird method (D+L), was utilized if there was heterogeneity based on Q-test p value less than 0.1. The significance of pooled ORs was tested by Z test (P<0.05 was considered significant).

Sensitivity analysis was performed, in which the meta-analysis estimates were computed after every one study being omitted in each turn.

Finally, publication bias was assessed by performing funnel plots qualitatively, and estimated by Begg's and Egger's tests quantitatively.

## Results

### Literature Search and Study Selection

After comprehensive searching, a total of 261 articles in English and 8 in Chinese were retrieved. In our meta-analysis were initially included altogether 19 studies [16]–[34] which catered to the inclusion criteria. Those 19 studies were preliminarily appropriate to the meta-analysis of the associations with gastric cancer regarding IL-8 -251 A/T SNP. After careful reading of the full text of those studies, we found two studies investigated by seemingly different but actually almost the same authors [32], [34], so we only included the study with larger sample size (34), that is, the study with smaller sample size [32] was finally excluded. The surnames and names of the authors [34] were rectified, that is, Bo S. et al were changed into Song B. et al for correct citation.

Traditionally speaking, any study that deviated from HWE should have been removed; however, Minelli C et al. recently pointed out that studies that appear to deviate from HWE should be investigated further rather than just excluded unless there are other grounds for doubting the quality of the study [44]. To date, it is still inconclusive whether studies deviated from HWE should be included or excluded in conducting meta-analysis. In our meta-analysis, one study [21] was deviated from HWE; however, considering that the number of participants in this study was large and given that sensitivity analyses would be conducted, we finally remained this study in our meta-analysis.

**Figure 1:**
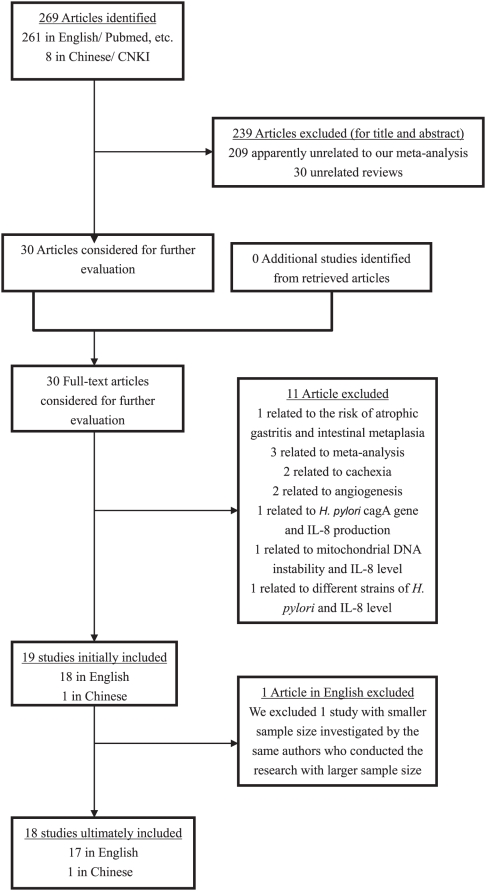
The flow chart of literature search and study selection.

Thus, 18 studies [16]–[31], [33], [34] with a total of 6554 controls and 4163 cases were ultimately eligible for the meta-analysis of IL-8 -251 A/T SNP. The corresponding characteristics were seen in Table 1. The flow chart of literature search and study selection was illuminated in Figure 1.

**Table 1:** Study Characteristics of genotypes in gastric cancer cases and controls in the analysis of Interleukin-8 -251 promoter polymorphism.

First author	Year of publication	Quality assessment scores	Genotyping method	Total sample size	Number of controls	Number of cases	Study location	Ethnic group	P values for HWE	Controls,genotypes(n)			All Cases,genotypes(n)		
										TT	TA	AA	TT	TA	AA
Savage SA et al.#	2004	7	Multiplex	517	429	88	China	Asians	0.8848131	147	207	75	26	39	23
Lu W et al.	2005	6.5	DHPLC	550	300	250	China	Asians	0.5158484	119	144	37	94	102	54
Taguchi A et al.# ∧ * ¶	2005	7	RFLP	648	252	396	Japan	Asians	0.9940137	125	105	22	161	191	44
Lee WP et al.# ∧ * ¶ +	2005	6	RFLP	778	308	470	Taiwan China	Asians	0.1430368	108	138	62	198	213	59
Ohyauchi M et al.∧ ¶ ★	2005	7.5	Direct	456	244	212	Japan	Asians	0.8470500	149	84	11	93	106	13
Zeng ZR et al.+	2005	6.5	RDB	402	196	206	China	Asians	0.0218768	43	114	39	37	110	59
Kamali-Sarvestani E et al.	2006	5.75	ASO	172	153	19	Iran	Asians◊	0.7975756	57	74	22	4	6	9
Shirai K et al.∧	2006	5.5	RFLP	649	468	181	Japan	Asians	0.8304604	211	208	49	83	78	20
Savage SA et al.	2006	7.5	Taqman/MGB Eclipse	715	428	287	Poland	Caucasians	0.3914650	106	205	117	71	140	76
Kamangar F et al.∧ ¶	2006	6	MGB Eclipse/Taqman	319	207	112	Finland	Caucasians	0.0549341	72	111	24	42	56	14
Garza-Gonzalez E et al.∧ * ¶	2007	3.75	ARMS	285	207	78	Mexico	Hispanic⊿	0.4918145	68	97	42	15	47	16
Crusius JB et al.# ∧	2008	8.5	Real-time PCR	1375	1139	236	European	Caucasians	0.7055677	315	574	250	75	113	48
Canedo P et al.	2008	6.5	Taqman	1026	693	333	Portugal	Caucasians	0.4597191	203	353	137	111	169	53
Ye BD et al.∧ ¶	2009	5.75	RFLP	359	206	153	Korea	Asians	0.5529341	97	86	23	54	82	17
Kang JM et al.* ¶ +	2009	7	RFLP	656	322	334	Korea	Asians	0.2256995	147	148	27	126	159	49
Ko KP et al.	2009	7	SNaPshot	389	308	81	Korea	Asians	0.1553548	135	146	27	34	35	12
Zhang L et al.# +	2010	8.5	RFLP	1023	504	519	China	Asians	0.7541397	160	251	93	130	261	128
Song B et al.	2010	5.5	RFLP	398	190	208	China	Asians	0.3894032	68	96	26	64	108	36

# Data of cardia type of gastric cancer were accessible;

∧ Data of noncardia type of gastric cancer were accessible;

*Data of sporadic diffuse-type of gastric cancer were accessible;

¶ Data of intestinal-type of gastric cancer were accessible.

+ Data of the status of *Helicobacter pylori* of gastric cancer were accessible.

◊ Here participants, though treated as Asians geographically in this study, should be better considered as separate Iranian population conducted in our subgroup analysis due to its complex ethnic components.

⊿ Here participants in this study should be treated as Mexican or Hispanic population rather than Caucasian population [26], [45].

★ Here control 1 in that study was selected as the control group in our meta-analysis because the gastric cancer participants were the same and control 2 was only used for further validation of the findings [20]. RFLP: Restriction fragment length polymorphisms; DHPLC: PCR-based denaturing high-performance liquid chromatography; Taqman: TaqMan polymerase chain reaction method; SSCP: Single strand conformation polymorphism; Multiplex: Multiplex polymerase chain reaction method (a variant of PCR in which two or more loci are simultaneously amplified in the same reaction); Direct: Direct sequence analysis of polymerase chain reaction; RDB: polymerase chain reaction and reverse dot blot; ASO: oligonucleotide allele specific polymerase chain reaction; MGB Eclipse: MGB Eclipse Assay polymerase chain reaction method; ARMS: Amplification refractory mutation system polymerase chain reaction; SNaPshot: the SNaPshot assay which provides detection of certain SNPs.

### Overall Meta-analysis and Subgroup Analyses

**Figure 2:**
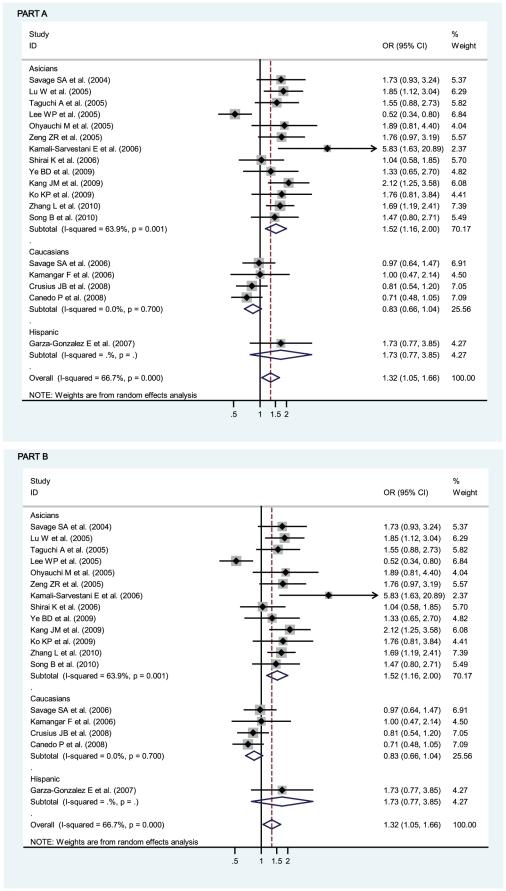
Odds ratios (ORs) for associations between IL-8 -251 A/T SNP and gastric cancer risk among different ethnicities, in order of increasing publication year, 2004–2010. Studies were entered into the meta-analysis sequentially by year of publication. The sizes of the squares indicate the relative weight of each study. Weights were derived from random-effects analysis. Bars, 95% confidence interval (CI). A) The IL-8 -251 AA genotype versus TT genotype; B) The IL-8 -251 AA genotype versus TA genotype.

**Figure 3:**
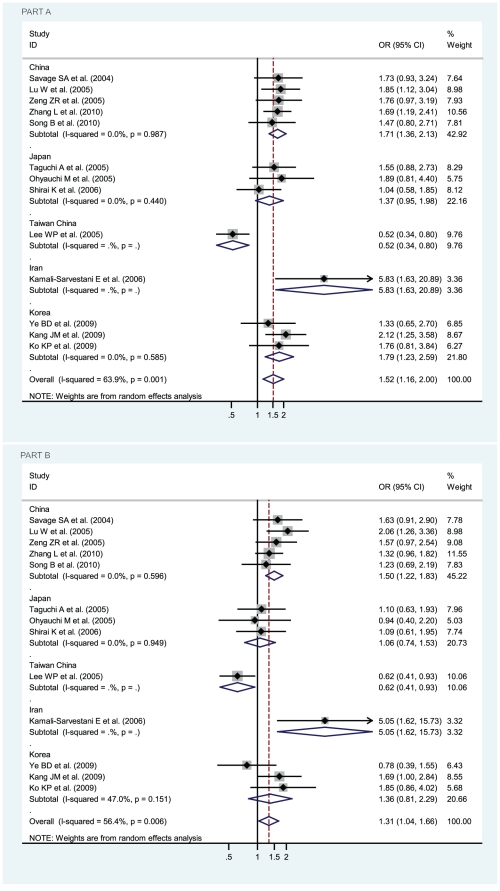
Odds ratios (ORs) for associations between IL-8 -251 A/T SNP and gastric cancer risk among geographically Asian participants from China, Taiwan China, Japan, Korea, and Iran. The sizes of the squares indicate the relative weight of each study. Bars, 95% confidence interval (CI). A) The IL-8 -251 AA genotype versus TT genotype; B) The IL-8 -251 AA genotype versus TA genotype.

OR_1_ (p value), OR_2_ (p value), and OR_3_ (p value) of IL-8 -251 A/T SNP were 1.32 (p = 0.018), 1.12 (p = 0.082), and 1.17 (p = 0.092), respectively, possibly insinuating a codominant model effect of putative susceptible A allele (OR_1_>OR_2_>1 and OR_1_>OR_3_>1). To further determine whether the adoption of codominant genetic model is influenced by the study deviated from HWE [21], the recalculated OR_1_ (p value), OR_2_ (p value), and OR_3_ (p value) of IL-8 -251 A/T SNP, after the study [21] had been removed, became 1.30 (p = 0.032), 1.13 (p = 0.094), and 1.15 (p = 0.155), respectively, also possibly insinuating a codominant model effect of putative susceptible A allele. Considering that the participants in the study [21] were Asians, we calculated OR_1_ (p value), OR_2_ (p value), and OR_3_ (p value) of IL-8 -251 A/T SNP among Asian participants, with both the inclusion and the exclusion of the study [21], and their values became 1.52 (1.16–2.00, p = 0.003), 1.19 (1.02–1.38, p = 0.023), and 1.31 (1.04–1.66, p = 0.024) when the study [21] was included and 1.51 (1.12–2.02, p = 0.006), 1.19 (1.02–1.39, p = 0.029), and 1.29 (1.00–1.67, p = 0.050) when the study [21] was excluded, definitely indicating a codominant model effect of putative susceptible A allele among Asians (OR_1_>OR_2_>1 and OR_1_>OR_3_>1 with almost all their p values statistically significantly less than 0.05 or one just reached 0.05). Thus, the inclusion of the study with deviation from HWE [21] does not influence the adoption of the most probable genetic model (codominant model) in our meta-analysis. The genotype AA was compared with the genotype TA (AA vs TA) and with the genotype TT (AA vs TT), respectively. In Figure 2, for overall gastric cancer no statistically significant finding could be observed (AA vs TA), whereas a statistically significant finding could be noted (AA vs TT) from the facts that the pooled OR (95% CI, p value) was 1.17 (0.98–1.40, p = 0.092) for the former but 1.32 (1.05–1.66, p = 0.018) for the latter. The data were stratified, in the light of the ethnicity of participants, into Caucasians, Asians, and Hispanic. Also in Figure 2, the apparently opposite tendency could be noted between Caucasians and Asians, and statistically significant findings were noted in Asians but not in Caucasians (AA vs TT). The pooled ORs (95% CIs, p value) were 1.52 (1.16–2.00, p = 0.003) and 0.83 (0.66–1.04, p = 0.100) in Asians and Caucasians (AA vs TT) or 1.31 (1.04–1.66, p = 0.024) and 0.93 (0.76–1.13, p = 0.453) in Asians and Caucasians (AA vs TA), respectively. Although the pooled OR could not be appraised in Hispanic participants, among which only one study was conducted in that ethnicity [26], the ethnicity that should be treated as Mexican or Hispanic rather than Caucasian [26], [45], the individual OR was still apparent, being 1.73 (0.77–3.85, p = 0.182) and 0.79 (0.40–1.54, p = 0.484) in Figure 2. We further sub-stratified Asians into the participants from China, Taiwan China, Japan, Korea, and Iran. As in Figure 3, the apparently discrepant tendency could be noted in the study from Taiwan China [19], the individual OR (95% CIs, p value) of which was 0.52 (0.34–0.80, p = 0.003); whereas the similar tendency could be noted in the studies from China, Japan, and Korea, the ORs (95% CIs, p value) of which were 1.71 (1.36–2.13, p = 0.000), 1.37 (0.95–1.98, p = 0.087), and 1.79 (1.23–2.59, p = 0.002), thus indicating statistically significant findings of increased risk for participants in China or Korea (AA vs TT). Likewise, similar findings were observed in the mode (AA vs TA), with 1.50 (1.22–1.83, p = 0.000), 1.06 (0.74–1.53, p = 0.743), 1.36 (0.81–2.29, p = 0.246), and 0.62 (0.41–0.93, p = 0.023) in China, Japan, Korea, and Taiwan China, respectively. Also interestingly, statistically significant finding was even more apparently noted in Iran because the individual OR for Iran (95% CIs, p value) was 5.83 (1.63–20.89, p = 0.007) and 5.05 (1.62–15.73, p = 0.005) in the mode (AA vs TT) and mode (AA vs TA), respectively.

As shown in Table 2 and Table 3, specific data for IL-8 -251 A/T SNP were stratified, on the basis of sample size, into two subgroups: large sample (the total number of controls and cases not less than 500) and small-and-moderate sample (the total number of controls and cases less than 500) subgroups. Statistically significant finding was noted in small-and-moderate sample subgroup but not in large sample counterpart (AA vs TT), given that the pooled ORs (95% CIs, p value) were1.62 (1.25–2.10, p = 0.000) for the former and 1.16 (0.86–1.57, p = 0.340) for the latter, respectively.

**Table 2:** Stratification for the test of heterogeneity on IL-8 -251 AA vs TA based on a codominant model.

	Q-test			OR(95%CI)	P value
	chi-squared	d.f.	p		
Overall	35.96	17	0.005	1.17(0.98–1.40)	0.092
Large sample	23.18	9	0.006	1.12 (0.90–1.40)	0.305
Small-and-moderate sample	11.75	7	0.109	1.25 (0.98–1.59)	0.067
High quality	17.88	10	0.057	1.25 (1.03–1.51)	0.023
Low-and-moderate quality	14.28	6	0.027	1.03 (0.71–1.49)	0.892
Publication before or in 2005	17.20	5	0.004	1.23(0.82–1.85)	0.326
Publication after 2005	18.40	11	0.073	1.13 (0.93–1.37)	0.222
Caucasians	0.98	3	0.806	0.93 (0.76–1.13)*	0.453#
Asians	27.54	12	0.006	1.31 (1.04–1.66)	0.024
Non-cardia type	4.56	7	0.714	0.85 (0.70–1.04)*	0.122#
Cardia type	4.77	4	0.311	1.22 (0.97–1.55)*	0.092#
Intestinal type	4.96	6	0.549	1.08 (0.83–1.40)*	0.583#
Diffuse type	6.42	3	0.093	0.89 (0.52–1.53)	0.672
Hp positive	14.11	3	0.003	1.22 (0.62–2.43)	0.564
Hp negative	2.43	2	0.297	0.88 (0.57–1.38)	0.589
PCR-RFLP genotyping	12.46	6	0.052	1.07 (0.82–1.41)	0.607
Other genotyping	23.23	10	0.010	1.25 (0.97–1.61)	0.084

*: M-H ORs (95% CI), otherwise D+L ORs (95% CI).

# : P values of M-H estimates, otherwise P values of D+L estimates.

**Table 3:** Stratification for the test of heterogeneity on IL-8 -251 AA vs TT based on a codominant model.

	Q-test			OR(95%CI)	P value
	chi-squared	d.f.	p		
Overall	51.03	17	0.000	1.32 (1.05–1.66)	0.018
Large sample	38.78	9	0.000	1.16 (0.86–1.57)	0.340
Small-and-moderate sample	6.08	7	0.530	1.62 (1.25–2.10)*	0.000#
High quality	27.54	10	0.002	1.38 (1.07–1.78)	0.013
Low-and-moderate quality	20.36	6	0.002	1.23 (0.77–1.97)	0.388
Publication before or in 2005	22.46	5	0.000	1.39(0.85–2.28)	0.191
Publication after 2005	28.50	11	0.003	1.28 (0.99–1.66)	0.063
Caucasians	1.42	3	0.700	0.83 (0.66–1.04)*	0.100#
Asians	33.20	12	0.001	1.52 (1.16–2.00)	0.003
Non-cardia type	19.22	7	0.008	1.05 (0.73–1.51)	0.783
Cardia type	12.12	4	0.016	1.20 (0.72–2.00)	0.481
Intestinal type	7.00	6	0.321	1.37 (1.05–1.79)*	0.021#
Diffuse type	12.14	3	0.007	1.24 (0.57–2.70)	0.595
Hp positive	22.19	4	0.000	1.56 (0.76–3.21)	0.230
Hp negative	4.88	3	0.181	0.99 (0.59–1.65)	0.967
PCR-RFLP genotyping	24.26	6	0.000	1.28 (0.86–1.89)	0.223
Other genotyping	26.45	10	0.003	1.34 (1.00–1.80)	0.048

*: M-H ORs (95% CI), otherwise D+L ORs (95% CI).

# : P values of M-H estimates, otherwise P values of D+L estimates.

The data were also stratified, in accordance with the quality appraisal scores, into high quality (scores no less than 6.5) and low-and-moderate quality (scores less than 6.5) subgroups. A statistically significant finding was witnessed in high quality subgroup but not in low-and-moderate quality counterpart, given that the pooled ORs (95% CIs, p value) were 1.38 (1.07–1.78, p = 0.013) for the former and1.23 (0.77–1.97, p = 0.388) for the latter (AA vs TT), and 1.25 (1.03–1.51, p = 0.023) for the former and 1.03 (0.71–1.49, p = 0.892) for the latter (AA vs TA), respectively.

The data were additionally stratified, in line with publication time, into the earlier publication subgroup (articles published before or in 2005) and the later publication subgroup (articles published after 2005). No statistically significant findings were observed on the grounds that the pooled ORs (95% CIs, p value) were 1.39 (0.85–2.28, p = 0.191) in the former and 1.28 (0.99–1.66, p = 0.063) in the latter (AA vs TT), and 1.23 (0.82–1.85, p = 0.326) in the former and 1.13 (0.93–1.37, p = 0.222) in the latter (AA vs TA), respectively.

When gastric cancer was classified into non-cardia (or distal) and cardia subtypes, no statistically significant findings were found among non-cardia type or among cardia type on the grounds that the pooled ORs (95% CIs, p value) were 1.05 (0.73–1.51, p = 0.783) among non-cardia type and 1.20 (0.72–2.00, p = 0.481) among cardia type (AA vs TT), and 0.85 (0.70–1.04 p = 0.122) among non-cardia type and 1.22 (0.97–1.55, p = 0.092) among cardia type (AA vs TA).

In terms of pathology, gastric cancer could be classified into intestinal, diffuse, or mixed subtypes, and a statistically significant finding was observed in intestinal-type cancer but not in diffuse-type cancer (AA vs TT), for the pooled ORs (95% CIs, p value) were 1.37 (1.05–1.79, p = 0.021) in the former and 1.24 (0.57–2.70, p = 0.595) in the latter (AA vs TT).

**Figure 4:**
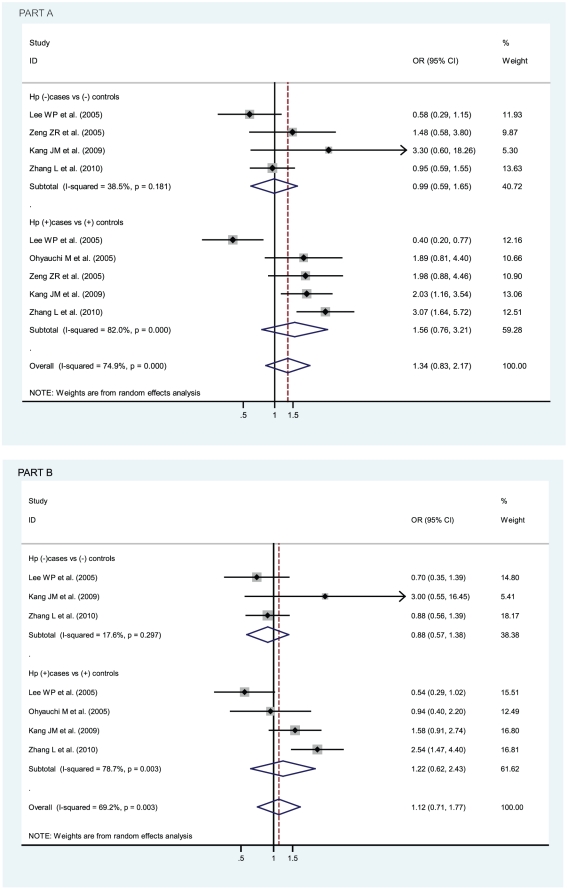
Odds ratios (ORs) for associations between IL-8 -251 A/T SNP and gastric cancer risk with different *H pylori* infection status. *H pylori* infection status includes *H pylori* positive cases versus positive controls and *H pylori* negative cases versus negative controls. The sizes of the squares indicate the relative weight of each study. Bars, 95% confidence interval (CI). A) The IL-8 -251 AA genotype versus TT genotype; B) The IL-8 -251 AA genotype versus TA genotype.

In terms of *Helicobacter pylori* infection status, no statistically significant findings were found among *Helicobacter pylori* positive cancer patients (compared with *Helicobacter pylori* positive controls) or among *Helicobacter pylori* negative cancer patients (compared with *Helicobacter pylori* negative controls), for pooled ORs (95% CIs, p value) were 1.56 (0.76–3.21, p = 0.230) in the former and 0.99 (0.59–1.65, p = 0.967) in the latter (AA vs TT), and 1.22 (0.62–2.43, p = 0.564) in the former and 0.88 (0.57–1.38), p = 0.589) in the latter (AA vs TA), as shown in Figure 4.

And when genotyping techniques were considered, a statistically significant finding was noted in other genotyping technique subgroup but not in traditional PCR-RFLP subgroup (AA vs TT). In the PCR-RFLP subgroup and in other genotyping technique subgroup, pooled ORs (95% CIs, p value) were 1.28 (0.86–1.89, p = 0.223) in the former and 1.34 (1.00–1.80, p = 0.048) in the latter (AA vs TT).

### Sensitivity Analysis

**Figure 5:**
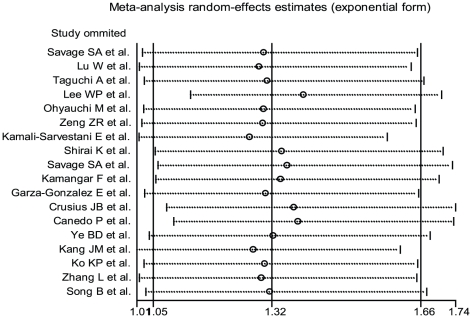
Influence analysis of the summary odds ratio coefficients on the association for the IL-8 -251 AA genotype with gastric cancer risk. Results were computed by omitting each study (on the left) in turn. Bars, 95% confidence interval. Meta-analysis random-effects estimates (exponential form) were used.

Meta-analyses were conducted repeatedly when each particular study had been removed. The results indicated that fixed-effects estimates and/or random-effects estimates before and after the deletion of each study were similar at large, suggesting high stability of the meta-analysis results. As shown in Figure 5, the most influencing single study on the overall pooled estimates seemed to be the study conducted by Lee WP et al. [19], the sensitivity analysis, however, indicated high stability of the results from the facts that the ORs (95% CI, p value) were 1.32 (1.05–1.66, p = 0.018) before the removal of that study and 1.39 (1.13–1.70, p = 0.002) after the removal of that study (AA vs TT).

### Cumulative Meta-analysis

**Figure 6:**
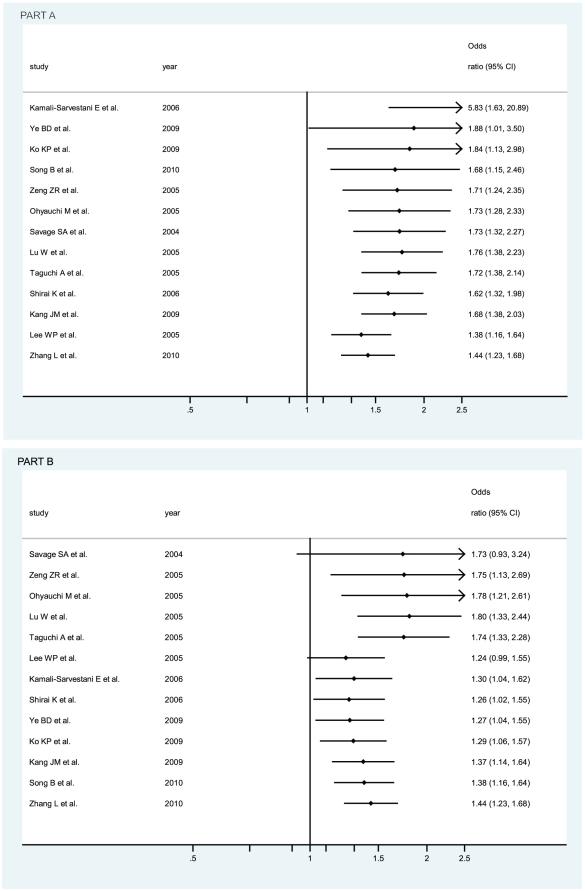
Cumulative meta-analysis of associations between the IL-8 -251 AA genotype, as compared with the TT genotype, and gastric cancer risk among Asians. Horizontal line, the accumulation of estimates as each study was added rather than the estimate of a single study. A) sorted primarily by total number of sample size; B) sorted primarily by publication time.

Cumulative meta-analyses of IL-8 -251 A/T SNP association were also conducted among Asians via the assortment of both total number of sample size (Figure 6 part A) and publication time (Figure 6 part B). As shown in Figure 6 part A, the inclinations, though undulated, toward significant associations could be seen when sorted by total sample size among Asians (AA vs TT). In Figure 6 part B was shown the cumulative meta-analysis of association for IL-8 -251 A/T SNP with overall gastric cancer among Asians in chronological order (AA vs TT).

### Publication Bias Analysis

**Figure 7:**
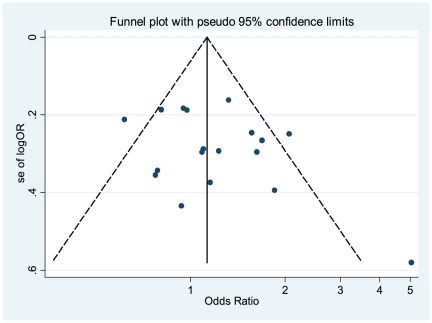
Funnel plot of publication bias for IL-8 -251 A/T SNP (AA vs TA). Note: Funnel plot with pseudo 95% confidence limits was used.

Publication bias was preliminarily examined by funnel plots qualitatively and estimated by Begg's and Egger's tests quantitatively. Its funnel plot (Figure 7) showed that dots nearly symmetrically distributed, predominantly within pseudo 95% confidence limits (AA vs TA). P values were 0.198 (AA vs TT) and 0.495 (AA vs TA) in Begg's test, insinuating no publication bias but p values were 0.031 (AA vs TT), insinuating a little publication bias but 0.171 (AA vs TA) in Egger's test, insinuating no publication bias.

## Discussion

In our meta-analysis, a statistically significant finding could be noted (AA vs TT) with the overall risk of developing gastric cancer; the apparently opposite tendency could be noted between Caucasians and Asians, and statistically significant findings were even more apparently noted in Asians, especially in Chinese Han population, but not in Caucasians (AA vs TT). Our meta-analyses suggest that IL-8 -251 AA genotype may be an important biomarker of gastric cancer susceptibility for Asians, especially for Chinese Han population, the assumption that needs to be further confirmed in future well-designed studies in China.

Based on the findings of cumulative meta-analyses, the inclinations, though undulated, toward significant associations in Asians could be obviously seen when sorted by total sample size (AA vs TT). The IL-8 -251 AA genotype may seem to be more susceptible to gastric cancer in Asians. Thus, the different or even conflicting risk associations, if so, among different ethnicities should be further meticulously investigated and reconfirmed in the future.

Our subgroup analyses also indicate that significant associations could be found in the small-and-moderate sample subgroup but not in the large sample counterpart (AA vs TT). In large sample subgroup the influences of ORs in the studies conducted by Lee et al. [19] and Kang et al. [30] are both oppositely strong enough (0.52 and 2.12, respectively) to offset the overall OR, thus the insignificant value (1.16) could be reached, whereas in small-and-moderate sample subgroup the ORs are averagely distributed around 1, but the influence of OR in the study conducted by Kamali-Sarvestani et al. [22] is strong enough (5.83) to make the overall OR to reach the significant value (1.62). A statistically significant finding was also witnessed in high quality subgroup but not in low-and-moderate quality counterpart (AA vs TT or AA vs TA). It is natural that high-quality studies should be designed in the future so as to accurately explore the real associations between IL-8 -251 A/T SNP and gastric cancer.

Additionally, 8 [18]–[20], [23], [25]–[27], [29] out of 18 eligible studies were dealt with noncardia gastric cancer and 5 [16], [18], [19], [27], [33] with cardia gastric cancer. No statistically significant findings could be noted with either subgroup. 7 studies [18]–[20], [25], [26], [29], [30] in our meta-analysis were dealt with pathologically intestinal-type gastric cancer and 4 [18], [19], [26], [30] out of 18 studies were dealt with pathologically diffuse-type gastric cancer. A statistically significant finding could be noted in intestinal-type but not in diffuse- type cancer (AA vs TT). As is known, cardia-type gastric cancer differs from noncardia-type gastric cancer in etiology, pathology, carcinogenesis, and/or prognosis [46]–[48], so is intestinal-type cancer versus diffuse-type cancer. It could be said that the indiscriminate combination of cardia-type and noncardia-type cases or intestinal-type and diffuse-type cases in the majority of eligible studies may mask or at least underestimate the real strength of the associations [5], [42].

Furthermore, *Helicobacter pylori* infection is associated with increased epithelial IL-8 expression and mucosal secretion of IL-8 and *Helicobacter pylori* induced IL-8 expression in gastric epithelial cells is associated *H pylori* with CagA positive phenotype [49]. In our meta-analysis, no significant associations could be found among *Helicobacter pylori* positive or negative cancer patients, which is inconsistent with the finding reported by Liu et al. [37]. The discrepancy could be explained that the study conducted by Lee et al. [19] was finally included in the *Helicobacter pylori* infection subgroup analysis in our meta-analysis but not in the meta-analysis by Liu et al. revealed in their Fig. 2 [37], because the OR in the study conducted by Lee et al. [19] is oppositely strong enough (0.40) to offset the overall OR, thus the insignificant value (1.56) could be reached. At any rate, the real association between *Helicobacter pylori* infection status and IL-8 -251 A/T SNP should be further meticulously investigated in the future.

With the advent of sophisticated genotyping technologies like seminested polymerase chain reaction, TaqMan allelic discrimination test, or real-time PCR, we may witness an upsurge of genetic association studies in the future. In our meta-analysis, statistically significant finding could be noted in other genotyping technique subgroups but not in conventional PCR-RFLP subgroup. The difference should be concerned with caution. We propose that the sensitivity and specificity of those genotyping techniques need to be further explored so as to seek out the optimal approaches which could minimize the genotyping errors [5], [42].

Up to now, two genome-wide association (GWA) studies related to gastric cancer have been published [50], [51]. They both reported that common variants associated with the risk of esophageal squamous cell carcinoma are also associated with the risk of cardia gastric cancer, but neither of them found IL-8 to be a risk gene of gastric cancer. Our explanation of the discrepancy is that the common initial stage of those two GWA studies focuses merely on esophageal squamous cell carcinoma. Therefore, albeit they found the shared risk variants between esophageal squamous cell carcinoma and gastric cancer, they might miss those risk genes which only confer risk to gastric cancer. Thus, we advocate more genetic studies, especially GWA studies, for gastric cancer to be carried out in the near future.

Finally, the strength of our meta-analysis could be summarized as follows. We sought to find as many publications as we could by means of various searching approaches. The study that appeared to deviate from HWE was not excluded mechanically in our meta-analysis unless there are other convincing grounds for doubting the quality of the study [44]. We laid more emphasis on assessing biases across studies and pinpointing the potential sources of heterogeneity via subgroup and sensitivity analyses. We comprehensively assessed the publication biases using several means like Begg's and Egger's tests as well as funnel plot tests. In view of this, we convince that the results of our meta-analysis, in essence, are sound and reliable.

Certainly, there are some unavoidable limitations in our meta-analysis. Firstly, the offered information from the included studies is inconsistent. Put it another way, the information about overall gastric cancer susceptibility is predominantly provided, while more important information about pathologic subtypes or anatomic subtypes of gastric cancer is less provided. Thus, the specific subtype results should be considered with caution. Secondly, with the merely published studies included in our meta-analysis, publication bias is very likely to occur, though no or a little statistically significant publication bias is indicated in our meta-analysis. Thirdly, moderate to severe heterogeneity could be witnessed among the included studies. So as to minimize the potential bias, we designed a rigorous protocol before conducting meta-analysis, and performed a scrupulous search for published studies using explicit methods for study selection, data extraction, statistical analysis, adoption of the most appropriate genetic model and sensitivity analysis.

In conclusion, IL-8 -251 AA genotype is associated with the overall risk of developing gastric cancer and may seem to be more susceptible to overall gastric cancer in Asian populations, especially for Chinese Han population. IL-8 -251 AA genotype is more associated with the intestinal-type gastric cancer. IL-8 -251 AA genotype is not associated with *Helicobacter pylori* infection status in our meta-analysis.

## Supporting Information

Table S1 **Scales for Quality Assessment.**
(DOC)
