# Supplementary material for: A Meta-Analysis of Interleukin-8 -251 Promoter Polymorphism Associated with Gastric Cancer Risk
Source: PLoS One. 2012 Jan 18;7(1):e28083. doi: 10.1371/journal.pone.0028083 (PMC3261138; doi:10.1371/journal.pone.0028083)
Supplement: Table S1 — Scales for Quality Assessment. (DOC) [file pone.0028083.s001.doc]

**Web Table 1** - Scales for Quality Assessment

| **Criteria** | **Scores** |
| --- | --- |
| **Credibility of controls** |  |
| Population - or neighborhood -based | 2.5 |
| Blood donors | 2 |
| Healthy volunteers with description | 1.5 |
| Healthy volunteers without description | 1 |
| Nongastroenterology division hospital-based patients | 0.5 |
| Gastroenterology division hospital-based patients | 0.25 |
| Not described | 0 |
| **Representativeness of cases** |  |
| Selection from some population cancer registry | 2 |
| Selection from some gastroenterology or surgery service | 1.5 |
| Selection with broad inclusion or exclusion criteria | 0.5 |
| Selection without any description in detail | 0 |
| **Consolidation of gastric cancer** |  |
| Both histopathologic and anatomic confirmation | 2.5 |
| Only histopathologic or anatomic confirmation | 2 |
| Only by medical record | 1 |
| Not described | 0 |
| **Genotyping examination** |  |
| Under "blinded" condition | 1 |
| Lacking "blinded" condition or not mentioned | 0 |
| **Association assessment** |  |
| Assessed association between genotypes and gastric cancer with appropriate statistics and examining confounders and effect modifiers (including anatomic location of cancer, and histology) | 0.5^*^2=1.0 |
|  |  |
